# The complete mitochondrial genome of soft coral *Sarcophyton trocheliophorum* (Cnidaria: Anthozoa) using next-generation sequencing

**DOI:** 10.1080/23802359.2019.1679677

**Published:** 2019-10-24

**Authors:** Chun-Yang Shen, Ya-Ting Dan, Alireza Asem, Pei-Zheng Wang, Wei Xue, Xiao-Bo Tong, Weidong Li

**Affiliations:** a Department of Biology, Chengde Medical University, Chengde, Hebei Province, China;; b College of Fisheries and Life Science, Hainan Tropical Ocean University, Sanya, China;; c Department of Chemical Engineering, Chengde Petroleum College, Chengde, China;; d Department of Physiology, Chengde Medical University, Chengde, Hebei Province, China

**Keywords:** Mitogenome, soft coral, *Sarcophyton trocheliophorum*, base composition, phylogenetic analysis

## Abstract

The complete mitochondrial genome of *Sarcophyton trocheliophorum* was completed using next-generation sequencing (NGS) method. The mitochondrial genome is a circular molecule of 18,508 bp in length, containing 14 protein-coding genes, two ribosomal RNA genes and one transfer RNA gene (Met-tRNA). The base composition is 30.45% A, 16.03% C, 19.13% G, and 34.40% T, with an A + T content of 64.85%. A phylogenetic analysis of Alcyoniidae showed that genus *Sarcophyton* had the closest relationship with *Sinularia*.

Species of soft coral genus *Sarcophyton* are widespread, from Polynesia in the east to the Red Sea in the west. Usually, they habitat in marine environment from the intertidal zone to depths up to 15 m. Colonies of *Sarcophyton* are characteristically fleshy and soft, mainly mushroom shape with yellow, beige, brown or green colour (Feussner and Waqa 2013).

Incomplete mitogenome of *Sarcophyton glaucum* is the only sequence in genus *Sarcophyton*, contains 11,715 bp (Beaton et al. 1998). In this study, we submitted and analyzed the first complete mitogenome of *Sarcophyton*, *Sarcophyton trocheliophorum* (GenBank: MK994517).

An individual of *S. trocheliophorum* was collected from the South China Sea (West Island, Sanya, Hainan province, China; 18°14′8.75″N, 109°22′39.10″E) and stored in Hainan Tropical Ocean University Museum of Zoology (NO.0001-St). The specimen was identified using *mtMutS* haplotype similarity. A genomic library was established followed by paired-end (2 × 150 bp) next-generation sequencing (10 Gb), using the Illumina HiSeq X-ten sequencing platform. The quality of produced sequencing reads was checked by FastQC (Andrews 2010). The sequences were assembled and mapped to the reference *Sinularia* mitochondrial genome (*Sinularia peculiaris*, JX023274) with Spades v3.9.0 (Bankevich et al. 2012) and bowtie v2.2.9 (Langmead and Salzberg 2012). Protein-coding genes (PCGs) and ribosomal RNA genes (rRNAs) were identified by alignment to the *Sinularia peculiaris* mitochondrial genome (GenBank: JX023274) and using online server NCBI ORF Finder (http://www.ncbi.nlm.nih.gov/gorf/gorf.html).

The determination of the putative transfer RNA gene (tRNAs) was performed by online software ARWEN (http://130.235.46.10/ARWEN/) and tRNAscan-SE2.0 (http://lowelab.ucsc.edu/tRNAscan-SE/).

The complete mitogenome of *S. trocheliophorum* was 18,508 in length, with a nucleotide composition of 30.45% A, 16.03% C, 19.13% G and 34.40% T.

The structure of *S. trocheliophorum* mitogenome was significantly different from classic metazoan mitogenomes, which contain 13 PCGs, 2 rRNAs and 22tRNAs.

The gene content and gene order in present mitogenome are the same as in the other Alcyoniidae, which include 14 PCGs, 2 rRNAs and 1 tRNA. Twelve genes (*cox1, 12S, nad1, cytb, nad6, nad3, nad4L, mutS, 16S, nad2, nad5*, and *nad4*) were located on the heavy strand and the other five genes (*tRNA-Met, cox3, atp6, atp8* and *cox2*) were encoded on the light strand. All PCGs were detected to start with the ATG codon. Seven genes (*nad1, nad6, nad3, nad2, nad5, cox*3 and *cox2*) appeared to use TAG as stop codon, whereas six genes (*cytb, nad4L, mutS, nad4, atp6*, and *atp8*) use the stop codon TAA, and cox1 use no premature stop codon T. We found that there was only one tRNA (Met-tRNA) that can be folded into typical clover-leaf secondary structures. There were a total of 15 gaps between coding genes, the length of which ranged from 3 nucleotides to 112 nucleotides. A 13 nucleotide overlap existed between nad2 and nad5, which was the same as *Sinularia peculiaris*. The whole mitogenome possessed strong A + T content bias. A + T content of the overall mitogenome was 64.85%, and A + T content of PCGs, rRNAs, and tRNA was 66.26, 58.7, and 56.34%, respectively.

A phylogenetic analysis of family Alcyoniidae was established based on 7 known Alcyoniidae mitogenomes (Beaton et al. 1998; Brockman and Mcfadden 2012; Kayal et al. 2013; Figueroa and Baco 2015; Shimpi et al. 2017; Asem et al. 2019) and an outgroup (*Chrysopathes Formosa*) (Brugler and France 2007). The concatenated dataset for nucleotides contained nine PCGs (published *S. glaucum* mitogenome lacks *nad3, nad4L, nad6, cytb*, and *mutS*) (Beaton et al. 1998). The maximum-likelihood (ML) phylogenetic analysis was performed based on the concatenated dataset by using the software MEGA X (Kumar et al. 2018). Regarding to phylogenetic tree, genera *Sarcophyton* and *Sinularia* revealed close evolutionary relationship (Figure 1).

**Figure 1.**
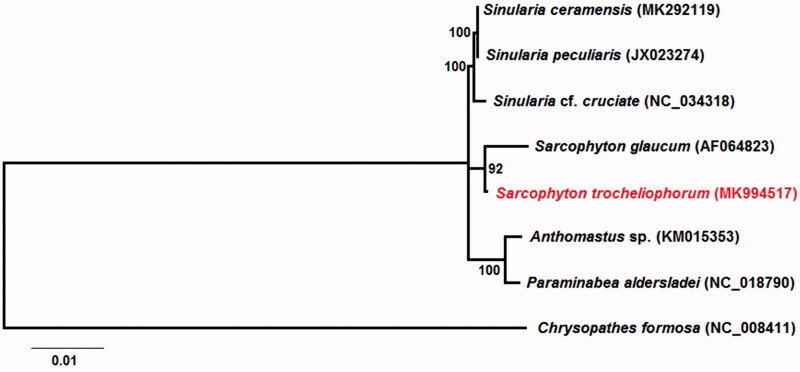
Phylogenetic tree of Alcyoniidae based on the concatenated nucleotides of nine protein coding genes and two rRNA genes using maximum-likelihood (ML). Numbers behind each node denote the bootstrap support values.
